# An entity that poses continuous challenge despite treatment attempt: lymphangioleiomyomatosis

**DOI:** 10.11604/pamj.2020.37.180.25608

**Published:** 2020-10-27

**Authors:** Munish Sharma, Salim Surani

**Affiliations:** 1 Department of Pulmonary Medicine, Corpus Christi Medical Center, Texas, USA,; 2 Texas A&M University, Texas, USA; 3 Pulmonary Medicine Fellowship Program, Corpus Christi Medical Center, Texas, USA

**Keywords:** Lymphangioleiomyomatosis, pneumothorax, sirolimus

## Image in medicine

In May of 2016, a 37-year-old Hispanic female, with no significant past medical history was brought to our hospital with right-sided chest pain. Chest X-ray revealed large right pneumothorax (A) and she underwent chest tube placement. Computed tomography (CT) chest without contrast showed innumerable cysts in the upper and lower lobes of bilateral lungs (B) and right thoracostomy tube with very small residual pneumothorax (C). CT abdomen showed hyperdense mass in left and possibly right kidney (D). CT head without contrast showed small calcifications in the lateral ventricles suggestive of tubers (E). A diagnosis of lymphangioleiomyomatosis (LAM) was suspected. Serum vascular endothelial growth factor D was < 800 pg/ml. She underwent open lung biopsy and diagnosis of LAM was established after histopathologic evaluation. Patient was sent to LAM clinic at a higher center and was started on sirolimus with an aim to stabilize her lung function, improve quality of life and functional performance. Since, sirolimus is used for suppressive rather than curative intent, patient has had 4 more episodes of spontaneous pneumothoaraces in last four years. Her last admission was in May of 2020 due to left sided secondary spontaneous pneumothorax (F), which completely resolved in 2 weeks without chest tube insertion (G).

**Figure 1:**
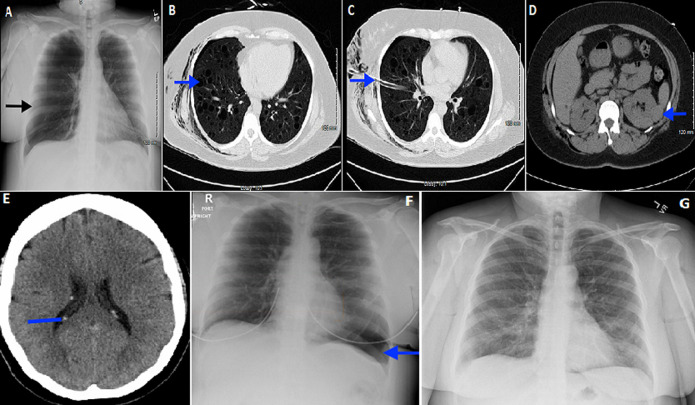
A) chest X-ray showing large right sided pneumothorax (arrow); B) CT chest without contrast showing innumerable cysts in bilateral lungs (arrow showing one of the cysts); C) CT chest without contrast showing right thoracostomy tube (arrow); D) CT abdomen showing hyperdense mass in left ( arrow); E) CT head without contrast showing small calcifications in the lateral ventricles suggestive of tubers ( arrow); F) chest X-ray showing left sided pneumothorax at lung base comprising approximately 30% of the thoracic volume; G) chest X-ray showing interval resolution of the left sided pneumothorax

